# The Use of Immunosuppressant Therapy for Multiple Sclerosis in Italy: A Multicenter Retroprospective Study

**DOI:** 10.1371/journal.pone.0157721

**Published:** 2016-06-27

**Authors:** Emanuele D’Amico, Carmela Leone, Giusi Graziano, Maria Pia Amato, Roberto Bergamaschi, Paola Cavalla, Gabriella Coniglio, Giancarlo Di Battista, Maria Teresa Ferrò, Franco Granella, Enrico Granieri, Alessandra Lugaresi, Giacomo Lus, Enrico Millefiorini, Carlo Pozzilli, Gioacchino Tedeschi, Mario Zappia, Giancarlo Comi, Maria Trojano, Vito Lepore, Francesco Patti

**Affiliations:** 1 MS center, Via S. Sofia, 78 95123, Catania, Italy; 2 Scientific Direction, National Cancer Institute "Giovanni Paolo II", Bari, Italy; 3 Department NEUROFARBA- University of Florence, Largo Brambilla 3, 50134, Florence, Italy; 4 CRISM, Istituto Neurologico Nazionale ‘C. Mondino’, Via Mondino 2, 27100, Pavia, Italy; 5 MS center, University of Turin, Via Cherasco, 15, Turin, Italy; 6 MS center, Hospital S. Carlo, Potenza, Italy; 7 MS center, Neurologia A.C.O. San Filippo Neri, Roma, Italy; 8 Hospital Maggiore, Crema, Italy; 9 Dipartimento di Neuroscienze, Via Volturno 39, Parma, Italy; 10 MS center, University of Ferrara, Ferrara. Italy; 11 MS center, University of Chieti, Chieti, Italy; 12 Clinica Neurologica I, Seconda Università di Napoli Ed.10 Policlinico Federico II, Via Sergio Pansini 5, 80131, Napoli, Italy; 13 Policlinico Umberto I, Rome, Italy; 14 University La Sapienza, Rome, Italy; 15 II University of Naples, Napoli, Italy; 16 HSR, University San Raffaele, Milano, Italy; 17 University of Bari Aldo Moro, Bari, Italy; National Natural Science Foundation of China, CHINA

## Abstract

**Introduction:**

Immunosuppressive agents (ISA) have been used in multiple sclerosis (MS) for decades, frequently as off label licensed therapies. Given the new MS treatment landscape, what place do ISA have in combating MS?

**Methods:**

We conducted a retrospective multicentre study to investigate the frequency of ISA prescription in 17 Italian MS centres, and to describe the clinical factors related to ISA use.

**Results:**

Out of 6,447 MS patients, 2,034 (31.6%) were treated with ISA, with Azathioprine being the most frequently used ISA overall. MS patients treated with ISA alone were more frequently affected by the progressive course (both primary and secondary) of the disease (RRR 5.82, 95% CI 4.14–8.16, p<0.0001), had higher EDSS (RRR 3.69, 95% CI 2.61–5.21, p<0.0001), higher assignment age (RRR 1.04, 95% CI 1.03–1.06, p<0.0001) than patients treated with only disease modifying drugs (DMDs).

**Conclusions:**

Progressive course, higher EDSS, higher assignment age were the strongest predictors of ISA prescription and use in our population.

## Introduction

The proper choice of a treatment represents a major challenge in the management of the patients with MS [1].

From the beginning of its description, MS pathogenesis has been ascribed to a deviation of the immune system [2]. Immunosuppressive agents (ISA) are inhibitors of crucial components of the immune system and they were used early in MS treatment; but their use was frequently off-label [3]. Two classes of immunomodulatory agents (DMDs) have been approved for the treatment of relapsing–remitting MS (RR-MS), that is interferon-β (INFs), and glatiramer acetate (GA) [4]. DMDs are able to shift immune responses from a pro-inflammatory toward an anti-inflammatory status; thus they were considered first-line options in MS management to modify the disease course in MS [4]. Later, mitoxantrone (an ISA), has been approved for treatment of active forms of RRMS and secondary progressive MS (SPMS) [5]. Furthermore, in the last years a number of oral ISA became, or are going to be, available in the MS management as first-line option [6].

The treatment landscape of MS is dramatically changing and the role of ISA in MS therapy scenario need to be deeply reconsidered and rewritten. Data about the frequency of their use were collected in the past, involving different countries worldwide; and the frequency of ISA prescription was found to be around 10% [7–8]. There were strong differences in frequency of ISA treatments per country [7–8]. To date we were unable to identify any large-scale clinical trials that characterize ISA prescription use in MS patients. Nonetheless, ISA continue to be used in MS patients, given that some patients are refractory to more conventional therapies or experienced contraindications to the use of more modern MS treatments such as with fingolimod or natalizumab. We designed and performed this study in Italy (involving the two principal regional areas) looking first at the frequency of ISA use. Moreover, we described the clinical features of MS patients who were ISA exposed to start questioning how and when it is possible thinking about ISA use in the clinical practice.

Our work could help the clinical neurologists to gain more insight in ISA prescription and use in MS therapy scenario and to consider how the use of ISA must be critically re-evaluated in the light of the recent changes in MS treatment landscape.

## Methods

A retrospective analysis of prospectively collected data was performed. The settings were referral subspecialty MS clinics in Italy. Twenty-five Italian MS centres (members of Italian iMEDWeb registry) were asked to participate in survey. Seventeen MS centres in the two different Italy’s regional area (eight in the North-Centre area, eight in the South-Sicily Island area) confirmed the participation in the study.

Clinical and demographical data of MS patients were recorded at each of the collaborating centers using the offline medical record iMED (Serono International SA, Geneva, Switzerland) and then uploaded to the Italian iMedWeb registry. The use of iMED as a research platform was approved by the local human research ethics committee at all participating centers. Quality assurance of clinical data was maintained by inbuilt data quality checking in the iMED local record system. To ensure consistency of Expanded Disability Status Score (EDSS) evaluations all neurologists completed the Neurostatus certification (http://www.neurostatus.net) or provided evidence of prior completion of this certification.

We searched through iMED software querying ISA and DMDs prescription. We identified two main groups of treated patients with MS: a group treated with ISA and a group treated with DMDs. The inclusion criteria were: i) diagnosis of MS according to Poser or McDonald criteria [9–10]; ii) disease duration of at least three years (evaluated as difference in time: last visit-onset disease); iii) at least three neurological evaluations performed by senior, trained neurologists in the MS diagnosis and management. We did not take in account patients being enrolled in experimental phase III studies. To avoid clinical misclassification we merged the secondary progressive and primary progressive patients below the umbrella definition of progressive forms, independently on their “activity status” [11]. ISA group included the patients treated at least one time with one or more of four drugs: azathioprine (AZA), cyclophosphamide (Cyc), methotrexate (MT) and mitoxantrone (MTX).

We divided the ISA group in three different subgroups: patients treated with ISA alone (arbitrarily defined as “*pure ISA”* therapy regimen), patients treated with ISA as first-therapy and then with DMDs (defined as *“induction”* therapy regimen) and patients treated with DMDs as first-therapy and then ISA (defined as *“escalation”* therapy regimen).

The fourth subgroup included DMDs patients, that is all MS patients treated with one or more of the different INFs formulations [Avonex® (Interferon beta-1a intramuscular once a week; Betaferon® (interferon beta-1b subcutaneous every second day); Extavia® (Interferon beta-1b subcutaneous every second day) Rebif^®^ (Interferon beta-1a, at dosage of 22 or 44 micrograms subcutaneous three times per week and Copaxone^®^ (Glatiramer Acetate subcutaneous every day)].

AZA was prescribed at variable doses ranging from 50 to 200 mg daily orally administered. Cyc was prescribed intravenously every month, 750–2000 mg per month with a cumulative dose of 4.800–30.000 mg per patient. MT was prescribed at doses of 7.5–15 mg weekly in 1–2 different oral administrations. MTX was prescribed intravenously at doses of 10–20 mg monthly for a cumulative dose of 60–200 mg.

The date of the enrollment visit was the date of the first therapy prescription and administration (ISA or DMDs). The EDSS [12] was recorded at baseline and at least every 3 months subsequently to determine the disability’s accrual.

**Ethics:** the study received the approval of the coordinating centre Ethical Committee (at Catania University). The participating centres informed their ethical committee, before making available informatics records of their patients at each centre. All of the recruited patients had signed an informed consent.

### Statistical analysis

Baseline characteristics of whole population were reported as frequencies and percentages for categorical variables and mean ± standard deviation (sd) for continuous variables. Disease duration was also reported as categorical variables. In particular, it was dichotomized according to clinical relevant cut off [13–15].

Patients were divided into four groups based on assigned therapy as previously indicated. Comparisons among multiple groups and associations with clinical parameters were performed with univariate multinomial logistic regression models. Multivariable analyses, with stepwise selection, were also conducted to investigate the effect of some confounding factors on the outcome of interest. Evaluated variables were those resulting significant at univariate analyses. The results were presented as relative risk ratios (RRR) with corresponding 95% confidence intervals and p-values. In a second step, the attention was focused on two main subgroups (all ISA vs DMDs). In this case simple and multiple logistic models were considered. Cochran-Armitage test was used to test for trend among proportions. The statistical significance was achieved at a p-value<0.05. All the analyses were performed using the Statistical Analysis System (SAS) Package, Release 9.2 (SAS Institute, Cary, NC, USA).

## Results

**Fig 1:**
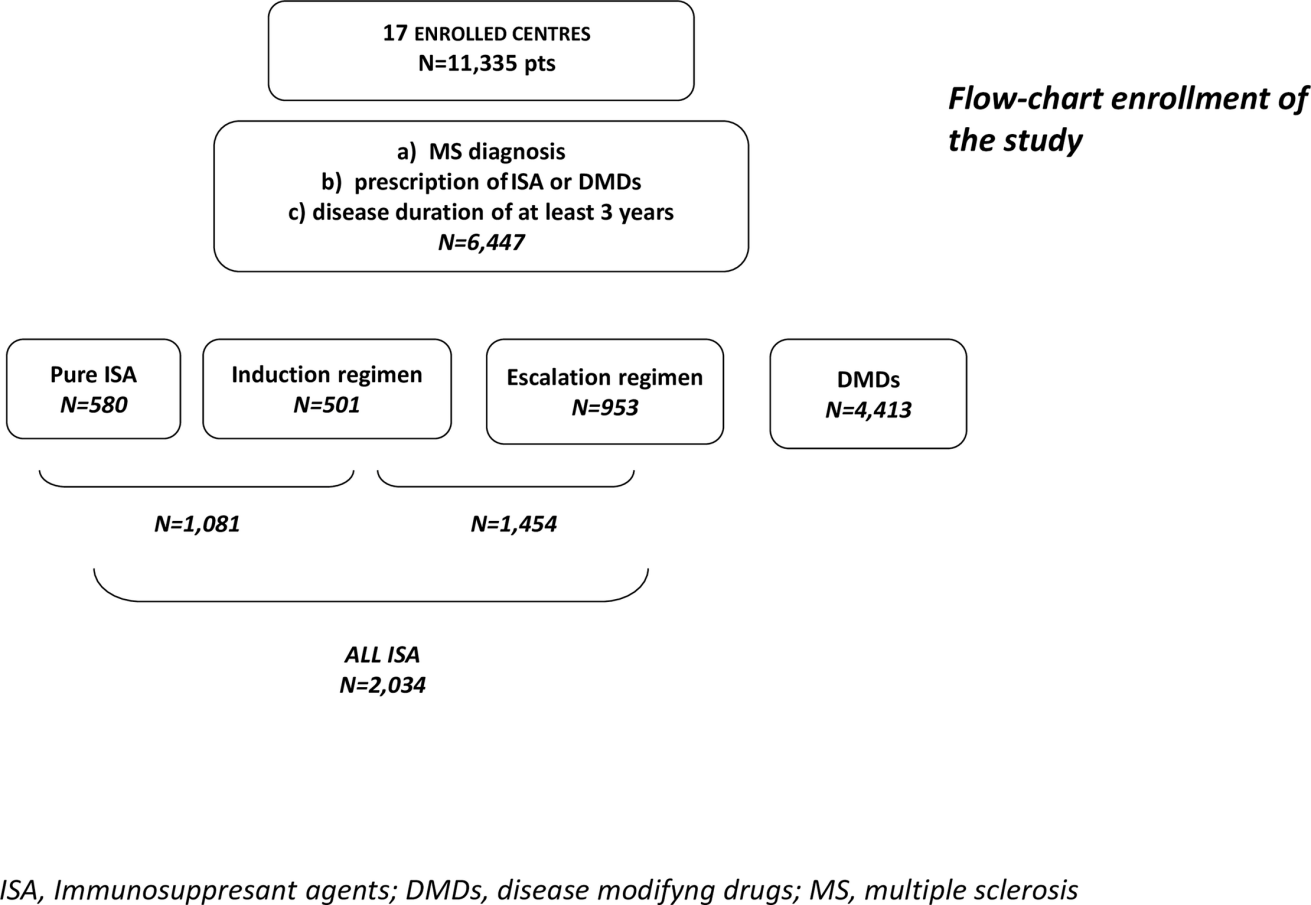
Shows the distribution of ISA subgroups in our cohort.

From a total sample of 11,335 MS patients, 6,447 (83%) patients were considered eligible for frequency analyses [S1 Table]. A number of 4,413 (68.4%) were treated with DMDs (INFs or GA) and 2,034 (31.6%) with ISA. Out of 2,034, 580 were classified as pure ISA, 501 were in induction scheme and 953 were in escalation scheme (Fig 1). Out of 2,034 ISA treated patients, AZA was the most frequently used in monotherapy (41.4%), followed by MTX (24,4%), then Cyc (7.9%) and MT (4.4%). The 21.9% of the whole group assigned to ISA treatment had used more than one single ISA drug.

A total of 4,624 patients were affected by RRMS and 1,006 suffered from progressive forms of MS (PFMS). The remaining 817 patients with MS were not yet clinical classified, because they had no the international used criteria [11–16] to be assigned to one of the two MS groups. EDSS evaluations were disposable in 4,202 patients and about 78% of patients had and EDSS value lower than 4.0. See Table 1.

**Table 1:** Patients’ characteristics at baseline.

Variable	Number (%) or mean±sd
**Number**	6,447
**Age at onset**	28.51±9.34
**Assignment age**	36±10.44
**Disease duration**	
*<5*	3,328 (82.13)
*≥5*	3,119 (48.38)
**Disease course**	
*RR*	4,624 (82.13)
*PP*	1,006 (17.87)
**EDSS**	
*<4*	3,291(78.32)
*≥4*	911(21.68)
**SEX**	
*M*	2,097 (32.53)
*F*	4,350 (67.47)
**Geographical areas**	
*Centre-North*	2,670 (41.41)
*South-Island*	3,777(58.59)

Table 2 shows the results of univariate multinomial logistic regression analyses. Regarding the age at MS onset, pure ISA subgroup had an older age, than DMDs and ISA subgroup (p<0.0001). Moreover, pure ISA group had significant higher assignment age than DMDs (p<0.0001). Regarding clinical characteristics, we found that pure ISA subgroup had longer disease duration (that is equal/more than five years) than DMDs. All ISA subgroups (pure, induction, escalation) experienced more frequently progressive course of the disease than DMDs (p<0.0001 for all). All ISA subgroups showed higher level of disability (EDSS value equal or higher of 4.0) (p<0.0001 for all) than DMDs group.

**Table 2:** Univariate multinomial logistic regression analyses.

Effect	n	Therapy (ref = DMDs)	RRR(95% CI)	p-value
**Age at onset**	580	*Pure ISA*	1.05(1.04–1.06)	<0.0001
	501	*Induction*	1.00(0.99–1.01)	0,339
	953	*Escalation*	0.99(0.99–1.01)	0,87
**Assignment age**	580	*Pure ISA*	1.08(1.07–1.09)	<0.0001
	501	*Induction*	1.01(0.99–1.02)	0,108
	953	*Escalation*	1(0.99–1.01	0,972
**Disease duration**	580	*Pure ISA*	2.59(2.15–3.12)	<0.0001
(= >5 vs <5)	501	*Induction*	1.05(0.87–1.26)	0,638
	953	*Escalation*	1.07(0.93–1.23)	0,358
**Disease course**	518	*Pure ISA*	15.87(12.89–19.54)	<0.0001
(PP vs RR)	435	*Induction*	5.22(4.16–6.55)	<0.0001
	759	*Escalation*	3.54(2.91–4.30)	<0.0001
**EDSS**	336	*Pure ISA*	14.28(11.07–18.43)	<0.0001
(= >4 vs <4)	195	*Induction*	4.81(3.56–6.48)	<0.0001
	515	*Escalation*	2.10(1.68–2.62)	<0.0001
**SEX**	580	*Pure ISA*	1.37(1.15–1.64)	0,0006
(M vs F)	501	*Induction*	1.29(1.06–1.56)	0,009
	953	*Escalation*	1.27(1.09–1.47)	0,002
**Geographical areas**	580	*Pure ISA*	1.68(1.39–2.02)	<0.0001
(South-Islands vs Centre-North)	501	*Induction*	1.05(0.87–1.26)	0,643
	953	*Escalation*	1.33(1.15–1.54)	0,0001

The multivariable multinomial logistic analyses confirmed the univariate results (Table 3).

**Table 3:** Multivariable multinomial logistic regression analyses.

Effect	Mean± SD or n (%)	Therapy (ref = DMDs)	RRR(95% CI)	p-value
**Assignment age**	44.14±12.02	*Pure ISA*	1.04(1.03–1.06)	<0.0001
	35.9±10.7	*Induction*	0.98(0.96–0.99)	0,006
	35.12±9.48	*Escalation*	0.99(0.98–1.00)	0,057
**Disease course**	315(60.81)	*Pure ISA*	5.82(4.14–8.16)	<0.0001
(PP vs RR)	147(33.79)	*Induction*	3.24(2.07–5.06)	<0.0001
	195(25.69)	*Escalation*	2.91(2.09–4.05)	<0.0001
**EDSS**	237(70.54)	*Pure ISA*	3.69(2.61–5.21)	<0.0001
(= >4 vs <4)	87(44.62)	*Induction*	3.33(2.21–5.02)	<0.0001
	134(26.02)	*Escalation*	1.57(1.16–2.12)	0,003
**Geographical areas**	397(68.45)	*Pure ISA*	1.45(1.06–1.97)	0,018
(South-Islands vs Centre-North)	288(57.49)	*Induction*	1.29(0.90–1.84)	0,168
	603(63.27)	*Escalation*	1.36(1.08–1.72)	0,009

Pure ISA subgroup had an older assignment age, more frequently PFMS and higher EDSS value than DMDs. Also induction and escalation ISA subgroups showed more frequently PFMS and higher EDSS value than DMDs. The findings were all statistically significant. We also found that in South-Island area there was higher frequency of pure and escalation ISA than DMDs compared with the Centre-North area (p<0.05 and <0.01, respectively).

**Fig 2:**
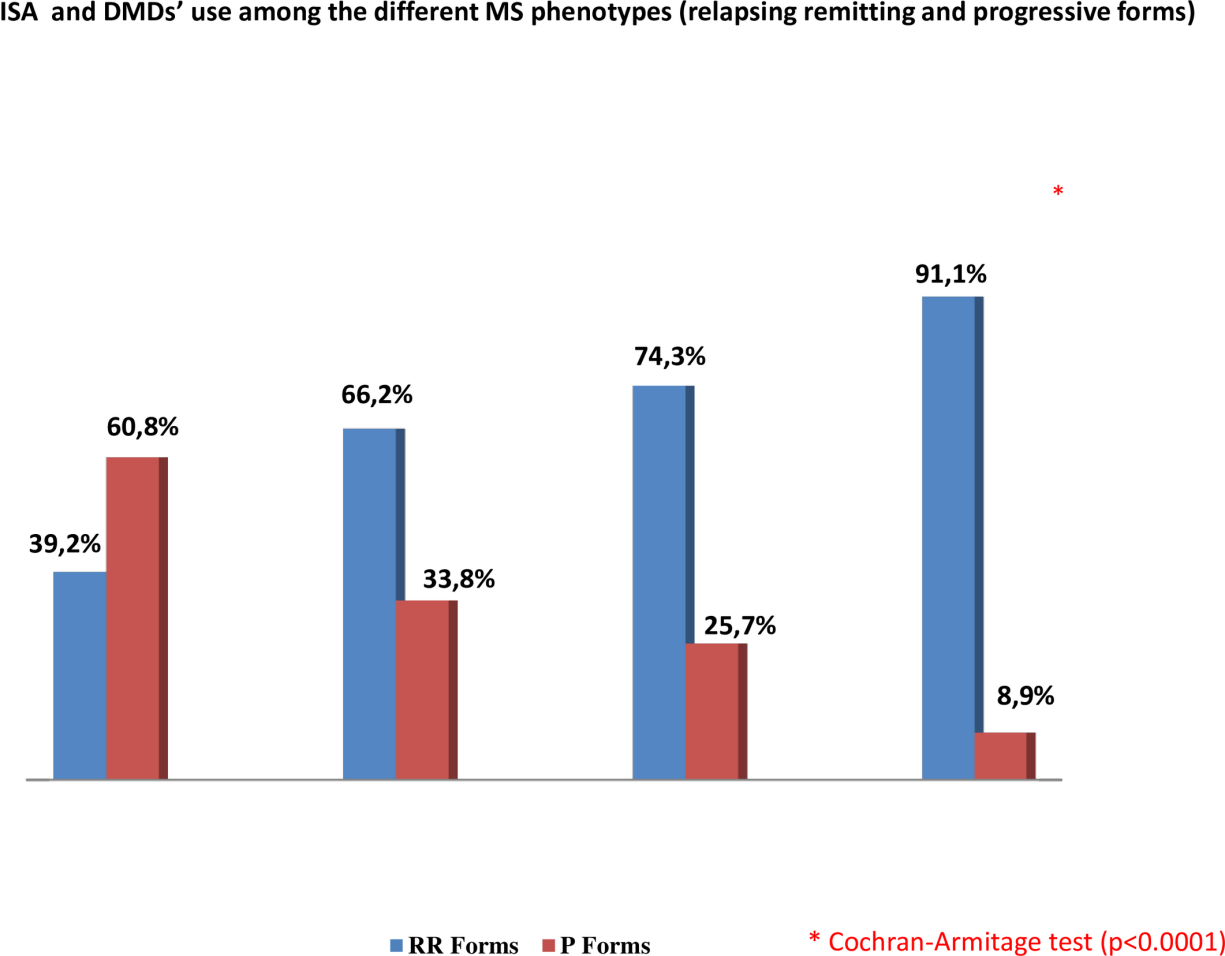
Shows the relation between the assigned therapy and the disease course in alla ISA subgroups and DMDs group.

Paying attention to the relation between the assigned therapy and the disease course it could be noticed that there was a decreasing percentage of progressive forms from pure ISA subgroup (the highest) to DMDs group (the lowest) (Fig 2). The trend showed in Fig 2 was confirmed by the Cochran-Armitage test (p<0.0001).

At the univariate logistic regression analyses (Table 4) all patients treated with ISA (pure, induction, escalation subgroups) were older at onset and at therapy assignment than DMDs group (p<0.0001). They also experienced longer disease duration (p<0.0001), PFMS (p<0.0001) and higher EDSS (p<0.0001). Furthermore these patients were more frequently men (p<0.0001) and settled in the South-Island area (p<0.0001). The multiple logistic regression confirmed only disease course, EDSS and geographical areas as independent predictors of ISA therapy (Table 5).

**Table 4:** Univariate logistic regression analyses performed on all ISA patients vs others.

Effect	mean±sd or n (%)	OR (95% CI)	p-value
**Age at onset**	29.46±10	1.016(1.010–1.022)	<0.0001
**Assignment age**	37.88±9.92	1.025(1.02–1.03)	<0.0001
**Disease duration**	1089(53.54)	1.35(1.22–1.50)	<0.0001
(= >5 vs <5)			
**Disease course**	657(38.38)	6.37(5.50–7.38)	<0.0001
(PP vs RR)			
**EDSS**	458(43.79)	4.65(3.97–5.44)	<0.0001
(= >4 vs <4)			
**SEX**	743(36.53)	1.30(1.16–1.45)	<0.0001
(M vs F)			
**Geographical areas**	1288(63.32)	1.34(1.20–1.49)	<0.0001
(South-Islands vs Centre-North)			

**Table 5:** Multivariable logistic regression analyses performed on all ISA patients vs others.

Effect	mean±sd or n (%)	OR(95% CI)	p-value
**Disease course**	657 (38.38)	4.03(3.19–5.09)	<0.0001
(PP vs RR)			
**EDSS**	458 (43.79)	2.44(1.97–3.01)	<0.0001
(= >4 vs <4)			
**Geographical areas**	1288(63.32)	1.37(1.14–1.64)	0,0007
(South-Islands vs Centre-North)			

## Discussion

About 30% of our MS patients had received at least one ISA prescription. MS patients who were older at age of therapy assignment and with higher disability (in terms of EDSS and PFMS course), have more frequently used ISA as pure ISA regimen. Moreover, pure ISA were more frequently prescribed in the South-Island area of our country, Italy. Previously, a worldwide survey about ISA prescription (including AZA, Cyc, MT, MTX) in 191 MS centres showed a mean percentage of ISA use about 10% [7]. Regarding Europe, France had the highest percentage (32.5%), and Italy had 12.7% of ISA use [7].

This study has potential limits, the first one is the retrospective design, although data were prospectively collected in each centre on patients who were regularly followed up. It also has the limit to miss data regarding patients without inclusion criteria (almost 3 years disease duration), or patients lost at follow-up (mainly because of moving to other centres). Moreover, the missing data in EDSS field, which did not invalidate the results.

In the last years, MS therapeutic armamentarium has dramatically grown up [17]. The introduction of new drugs with immunosuppressant profile has led the MS clinician to take in account an earlier and more aggressive approach in the MS treatment [17]. Nevertheless, the paucity of long term safety data, may have limited their use in the clinical practice. So, ISA may represent a useful therapeutic option to satisfy a new therapeutic approach aimed at a more aggressive and even earlier treatment with a well-known profile of toxicity.

In a recent non-inferiority trial 150 patients with RRMS were randomized to receive AZA 3 mg/kg daily (n = 77) or INF beta-1a 22 or 44 μg (n = 69) or INF beta-1b (n = 4) for two years. The intent-to-treat analysis was done on 68 and 63 patients, respectively. Annualized relapse rates were not significantly different between the AZA and INF-beta groups in the first (0.37 vs 0.47; p<0.05 and second (0.18 vs 0.29; p<0.05) years and, when combined for the two years, the annualized relapse rate was lower in the AZA group (0.26 vs 0.39; p<0.05 p = 0.07) [18]. In the AZA group there were more treatment discontinuations than in the INFs group because of adverse events [18]. These interesting data showed that AZA may be as effective as INFβ in RRMS as first-line therapy and shed new light on the possibility that MS physicians might use ISA as a suitable first line-drug to be prescribed. Obviously, such a strategy requires closer clinical monitoring during the first years, in particular to titrate the dose according to the lymphocyte count, and in general to manage all alerts reflecting a possible individual susceptibility. On the other hand, for some patients, ISA could provide a higher quality of life without the flu-like symptoms of INF or because of a less invasive route of administration, and with a known side effect profile, which could be easily monitored.

Progressive course of MS (both primary and secondary) and higher level of disability were correlated to overall ISA prescription in our study. These data are in line with previous reports about a higher ISA use in PFMS, that is in more impaired patients, and moreover, a preferred use as second-line therapy [8]. However, in our sample, the 39.2% of patients who had used ISA alone (pure ISA subgroup) did not have PFMS; and the percentage of patients with RRMS who had used ISA in escalation or induction was around 70%.

Two different therapeutic approaches are actually used in MS: escalation and induction therapy. Escalation therapy consists of starting, in the early phases of the disease, with first line DMDs (INFs, GA, teriflunomide, dimethyl fumarate) and if DMDs are ineffective or partially effective, a switch to second line drugs (MTX, natalizumab, fingolimod and other off-label used ISA as AZA, Cyc, MTX) and, in case of more failures, a third line options, such as alemtuzumab or autologous hematopoietic transplant could be considered[17]. Induction therapy consists of the early use of more aggressive therapy (such as ISA) followed by long-term maintenance treatment, generally with DMDs [19]. The use of natalizumab and fingolimod as first line drugs is indicated for aggressive forms of RRMS [17]. Both escalating and induction strategies can be successfully applied on the basis of clinical and radiological data; apart from clinically aggressive RRMS, for which induction treatment could be regarded as the first line of treatment. The use of more aggressive drugs in MS will make it particularly important to have long term safety data.

Both MTX and Cyc have the limit of cumulative doses [20–23]. Fewer concerns are raised by AZA; it was reported that long periods of treatment with this drug could expose patients to the risk of malignancies [24].

In the last decades, the economic burden of the disease has also received increasing attention, especially when considering efforts aimed to control public expense along with the decline of available resources for the healthcare services in Western countries [23]. Costs of MS are highly due to the chronic nature of the disease, the need for hospitalization during phases of severe relapses and to the gradual increase in disability, which may necessitate assistance with daily life activities.

In Italy, MS is the second CNS disorder for cost per patient, with an estimated cost of around €25,000 per MS patient/year; the costs of DMDs were significantly lower for patients with EDSS scores higher than 4.0 [25]. All of these reasons underline as the therapeutic decisions of the MS physician have to take into account the related economic weight. Probably, among the reasons of the prominent use of ISA in South-Island area of Italy, there was a different economic organization of the local healthcare system [25].

Several new drugs have recently demonstrated their efficacy in RRMS in randomized controlled phase III trials, and have been recently approved in some countries or their approval can be expected in the near future [26]. The future challenge will be to choose the most efficacious and safe drug for the individual patient. Biomarkers that help to predict these issues in the individual are mandatory [27]. The course of MS varies between individuals and intra-individual during the course of the disease: some patients accumulate minimal disability over their lives, whereas others experience a rapidly disabling disease course. This latter subset of patients show a rampant progression of disability over a short time period, and they are referred to have an 'aggressive' MS. Treatment of patients with aggressive MS is challenging, and optimal strategies have yet to be defined.

Given potentially severe side-effects of newer agents for the treatment of patients with MS, including monoclonal antibodies that possess immunosuppressive properties, it is likely that the established immunosuppressant approach will not be replaced in the therapy of MS in the near future. A selected group of highly active MS patients may benefit from a “hit hard and “early” approach, driven by the essential aim to suppress the inflammatory cascade which plays a crucial role in the early stage of the disease. It is clear that more investigations are needed to clarify the optional therapeutic strategies for MS treatment.

**Names of drugs:**Avonex® (Interferon beta-1a intramuscular once a week); Betaferon® (Interferon beta-1b subcutaneous every second day); Extavia® (Interferon beta-1b subcutaneous every second day); Rebif^®^ (Interferon beta-1a, at dosage of 22 or 44 micrograms subcutaneous three times per week, Copaxone^®^ (Glatiramer Acetate subcutaneous every day); Azathioprine; Metotrexate;Mitoxantrone; Cyclophosfamide; Tysabri® (natalizumab); Gylenia®(Fingolimod).

## Supporting Information

S1 Table This excel file shows the entire database of our multi center study. (XLS)
